# Functions of PUF Family RNA-Binding Proteins in *Aspergillus nidulans*

**DOI:** 10.4014/jmb.2101.01011

**Published:** 2021-03-12

**Authors:** Sung-Hun Son, Seo-Yeong Jang, Hee-Soo Park

**Affiliations:** 1 School of Food Science and Biotechnology, Kyungpook National University, Daegu 41566, Republic of Korea; 2 Department of Integrative Biology, Kyungpook National University; Daegu 41566, Republic of Korea

**Keywords:** PUF domain, RNA-binding protein, asexual development, sterigmatocystin, conidia

## Abstract

RNA-binding proteins are involved in RNA metabolism and posttranscriptional regulation of various fundamental biological processes. The PUF family of RNA-binding proteins is highly conserved in eukaryotes, and its members regulate gene expression, mitochondrial biogenesis, and RNA processing. However, their biological functions in *Aspergillus* species remain mostly unknown in filamentous fungi. Here we have characterized the *puf* genes in the model organism *Aspergillus nidulans*. We generated deletion mutant strains for the five putative *puf* genes present in the *A. nidulans* genome and investigated their developmental phenotypes. Deletion of *pufA* or *pufE* affected fungal growth and asexual development. *pufA* mutants exhibited decreased production of asexual spores and reduced mRNA expression of genes regulating asexual development. The *pufE* deletion reduced colony growth, increased formation of asexual spores, and delayed production of sexual fruiting bodies. In addition, the absence of *pufE* reduced both sterigmatocystin production and the mRNA levels of genes in the sterigmatocystin cluster. Finally, *pufE* deletion mutants showed reduced trehalose production and lower resistance to thermal stress. Overall, these results demonstrate that PufA and PufE play roles in the development and sterigmatocystin metabolism in *A. nidulans*.

## Introduction

*Aspergillus nidulans* is a model filamentous fungus widely used in fungal development and metabolism studies [1, 2]. *A. nidulans* propagates through both asexual and sexual spores, but asexual spores are their primary reproductive particle [3, 4]. Spores germinate to form germ tubes and a web-like mass of fungal hyphae [5]. These hyphae acquire developmental competence and undergo asexual or sexual development, depending on the environmental conditions [6, 7]. For example, light can induce the formation of asexual structures—the conidiophores—but sexual fruiting bodies—the cleistothecia—are formed in dark conditions [8, 9]. These reproductive processes are regulated by a myriad of transcription factors and several signaling pathways [10, 11]. In asexual development, BrlA, AbaA, and WetA are central transcription factors that control mRNA expression of development-related genes [6, 12]. BrlA is a C_2_H_2_ zinc-finger transcription factor that controls mRNA levels of *abaA* and other developmental genes [13]. AbaA induces mRNA expression of three key transcription factors: WetA, VosA, and VelB [14-16]. These transcription factors regulate asexual spore maturation, trehalose biosynthesis, and conidial stress tolerance [15, 17]. Fungal species produce various primary and secondary metabolites during development [18]. Secondary metabolites that have detrimental effects on humans are called mycotoxins [19, 20]. Sterigmatocystin produced by *A. nidulans* is a precursor aflatoxin and is considered as a group 2B carcinogen [21]. Sterigmatocystin biosynthesis depends on a gene cluster of which the AflR transcription factor is a key regulator [22-24]. Other important genes involved in sterigmatocystin production include MtfA, VeA, LaeA, and McrA [25-28].

RNA-binding proteins play essential roles in the posttranscriptional regulation of gene expression in eukaryotes [29-31]. In fungi, RNA-binding proteins participate in various fundamental processes, including development, stress responses, filamentation, and pathogenesis [32, 33]. The PUF (Pumilio/Fem-3 binding factor) family of canonical RNA-binding proteins is conserved among most eukaryotic systems [34]. The Pumilio in *Drosophila melanogaster* and Fem-3 binding factor (FBF) in *Caenorhabditis elegans* are the founding members of the PUF family proteins [35]. They contain several repeated PUF domains, which interact with sequence-specific RNA elements of their target. [35]. In Ascomycota fungi, PUF proteins recognize 8- to 10-nucleotide binding elements, reducing translational efficiency or by increasing its decay [36, 37]. Most of the research on the RNA targets and the biological roles of the PUF proteins have been carried out in *Saccharomyces cerevisiae* [35, 38, 39]. *S. cerevisiae* contains six PUF proteins involved in posttranscriptional processes, mating type switching, lifespan, thermotolerance, cell wall integrity, and mitochondrial biogenesis [35, 40]. For example, PUF6 targets mRNA of Ash1, which is involved in budding [41]. However, little is known about PUF proteins in filamentous fungi, particularly in *Aspergillus* species.

RNA-binding proteins, including SwoK, FabM, PbpA, and RrmA, have been characterized in *A. nidulans* [42-46]. These RNA-binding proteins regulate fungal development and primary and secondary metabolisms. In this study, we characterized the *A. nidulans* PUF family of RNA-binding proteins. The *A. nidulans* genome encodes five putative proteins containing PUF repeated motifs (PufA to E). With phenotypic and genomic analyses, we show that PufE is required for normal fungal development, spore maturation, and sterigmatocystin production in *A. nidulans*.

## Materials and Methods

### Strains, Media, and Growth Conditions

The *A. nidulans* strains used in this study are listed in Table 1. Fungal strains were grown on solid or liquid minimal media with 1% glucose (MMG) [47]. For auxotroph mutants, uridine (Acros Organics, USA), uracil (Acros Organics), and pyridoxine (Sigma-Aldrich, USA) were supplemented in the media. Sexual media (SM) was used to induce sexual development [48]. To observe developmental phenotypes, the strains were point inoculated in solid MMG or SM and cultured at 37°C for 5-7 days. For sterigmatocystin extraction, the strains were cultured in liquid complete media (CM) at 30°C for 7 days. To collect fresh conidia, each strain was incubated in solid MMG at 37°C for 2 days. For amplification of the plasmid used to generate the complemented strains, *Escherichia coli* DH5α (Enzynomics, Korea) cells were grown in a Luria–Bertani medium (BD Difco, USA) with ampicillin (100 μg/ml) (Sigma-Aldrich).

### Construction of *puf* Deletion Mutant Strains

The oligonucleotides used in this study are listed in Table 2. To generate deletion mutants for *puf* genes, disruption cassettes of each gene were prepared using the double-joint PCR method [49]. First, the 5’ and 3’ regions of each *puf* gene were amplified from the *A. nidulans* FGSC A4 genomic DNA template, using DF/TailR and DR/TailF primer pairs. The *A. fumigatus* *pyrG* (*AfupyrG*) was used as a selection marker and was amplified from the *A. fumigatus* genomic DNA template using a 5’ AfupyrG_F (OHS089)/3’ AfupyrG_R (OHS090) primer pair. The amplified 5’ and 3’ regions of the *puf* genes and the *AfupyrG* selection markers were linked through PCR and amplified using an NF/NR primer pair to obtain the *puf* gene disruption cassettes. The deletion cassette was introduced into protoplasts of the *A. nidulans* RJMP1.59 strain, made with the Vinoflow FCE lysing enzyme (Novozymes, Bagsvaerd, Denmark) [47]. After culturing transformed cells in the selection media (MMG without uridine and uracil), transformants were confirmed by PCR followed by restriction enzyme digestion.

### Generation of the Complemented *pufA* and *pufE* Strains

To generate complemented strains, the regions containing the promoter and open reading frame sequences of *pufA* or *pufE* were amplified with the OHS1136/OHS1137 or OHS1380/OHS1381 primer pairs, respectively, and digested with *Not*I. These DNA fragments were inserted into pHS13 [14], generating pSH4.1 or pSH5.1, for *pufA* or *pufE*, respectively. The pSH4.1 or pSH5.1 plasmid was introduced into the Δ*pufA* (TSH8.1) or Δ*pufE* (TSH27.1) strains to give rise to TSH17 and TSH28, respectively.

### Quantitative Reverse-Transcription PCR (qRT-PCR) Analysis

For qRT-PCR analysis, the samples were prepared as described previously [50]. Asexual spores of each strain were inoculated in liquid MMG and cultured at 37°C for 16 h. Cultured mycelia were squeeze-dried and stored at -80°C until RNA extraction. For the asexual developmental samples, the mycelia were filtered using Miracloth (Calbiochem, USA) and monolayered onto solid MMG. The plates were incubated for 12 and 24 h in light conditions to induce asexual development. After squeeze-drying, the samples were stored at −80°C until RNA extraction. For conidia, each strain was cultured in solid MMG at 37°C for 2 days and then the conidia were collected using Miracloth and stored at −80°C until RNA extraction.

Total RNA was extracted as described previously [50]. The samples were homogenized in a TRIzol reagent (Invitrogen, USA) and 0.5 mm zirconia/silica beads (RPI, USA), using a Mini-Bead Beater (BioSpec Product, USA). Then, the supernatant was separated by centrifugation, transferred into a new tube, and mixed with cold isopropanol. The pellets were resuspended with RNase-free water (Promega, USA) and treated with DNase I (Promega) to remove DNA. cDNA was synthesized with the GoScript Reverse Transcriptase (Promega). Quantitative PCR was conducted using iTaq Universal SYBR Green Supermix (Bio-Rad, USA) and CFX96 Touch Real-Time PCR (Bio-Rad). The expression levels for each target gene were calculated using the 2^-ΔΔCT^ method. The β-actin gene expression was used as an endogenous control.

### Quantification of Asexual spores and Cleistothecia

To test conidia production, the strains were inoculated in solid MMG at 37°C for 5 days, in light or dark conditions. Colony morphology was photographed with a Pentax MX-1 digital camera and a Zeiss Lab.A1 microscope equipped with an AxioCam 105c camera and AxioVision (Rel. 4.9) digital imaging software. For quantification, conidia were collected from the plate using ddH_2_O and Miracloth. The number of conidia was determined in triplicate for each strain with a hemocytometer.

For the formation of cleistothecia, each strain was point inoculated in solid SM and cultured in dark conditions at 37°C for 7 or 14 days. Cultured plates were washed with 100% ethanol, and the size of cleistothecia was measured using a Zeiss Lab.A1 microscope equipped with an AxioCam 105c camera and AxioVision (Rel. 4.9) digital imaging software. To measure the number of ascospores, cleistothecia were collected from each strain and broken into e-tubes. Then, they were diluted with ddH_2_O and measured with a hemocytometer. The number of ascospores was counted in triplicate for each strain.

**Sterigmatocystin (ST) Extraction and Thin Layer Chromatography (TLC) Analysis.** To extract ST from each strain, approximately 10^5^ conidia were inoculated into 5 ml liquid CM and cultured at 30°C for 7 days in dark conditions [51]. Then, 5 ml of chloroform was added to the cultured samples and the samples were mixed. The organic phases were separated through centrifugation, transferred to a new glass vial, and evaporated overnight. After complete evaporation, 200 μl of chloroform was added, and approximately 6 μl of the sample was loaded onto a TLC silica plate (Kiesel gel 60, 0.25 mm; Merck, Germany). Commercial sterigmatocystin (Sigma-Aldrich) was used as a loading control. To separate the samples, the TLC plate was developed in a chloroform: ethyl acetate (9:1 v/v) solution and treated with 1% aluminum hydroxide hydrate (Sigma-Aldrich). The TLC plate was observed under UV light (366 nm) and the relative intensity of the ST spots was calculated using ImageJ. The experiments were conducted in a triplicate for each strain.

### Trehalose Assay

The trehalose content in conidia was determined as previously described [52]. Briefly, the strains were incubated in solid MMG at 37°C for 2 days and approximately 2 × 10^8^ conidia were collected with ddH_2_O with 0.01% Triton X-100 (Sigma-Aldrich). After centrifugation, the supernatant was removed and resuspended in 200 μl of ddH_2_O with 0.01% Triton X-100 and heated at 95°C for 20 min. The samples were separated by centrifugation, and two equal volumes of supernatant per strain were transferred to a new tube. A volume of 0.2 M sodium citrate (pH 5.5) was added to the two prepared samples. Trehalase (3 mU, Sigma-Aldrich) was added to one of the two samples (the other served as the negative control), and samples were incubated at 37°C for 8 h to decompose trehalose into glucose. The amount of glucose present was measured using a Glucose Assay Kit (Sigma-Aldrich). The glucose assays of were done in triplicate for each strain.

### Spore Viability Assay

The method to test spore viability was described previously [51]. Briefly, strains were incubated in solid MMG at 37°C for 2 or 10 days; subsequently, approximately 10^3^ conidia were collected with ddH_2_O containing 0.01%Triton X-100 (Sigma-Aldrich). Approximately 100 conidia were spread onto solid MMG and cultured for 2 days at 37°C. Spore viability was measured by determining the number of colonies formed.

### Thermal Tolerance Assay

Thermal tolerance tests were performed as previously described [53]. Briefly, approximately 10^3^ conidia per ml were incubated at 55°C for 15 min or 30 min. Then, 100 μl of the conidia suspension was spread onto solid MMG. After incubation at 37°C for 2 days, the number of colony-forming units was counted. Conidia not treated with heat were used as a control. All experiments were carried out in triplicate.

### Statistical Analysis

Student’s unpaired *t*-tests were used to evaluate the statistical differences between control and deletion mutants. Data are reported as mean ± standard deviation. *P*-values < 0.05 were considered statistically significant.

## Results and Discussion

### *puf* Genes in *A. nidulans*

**Fig. 1:**
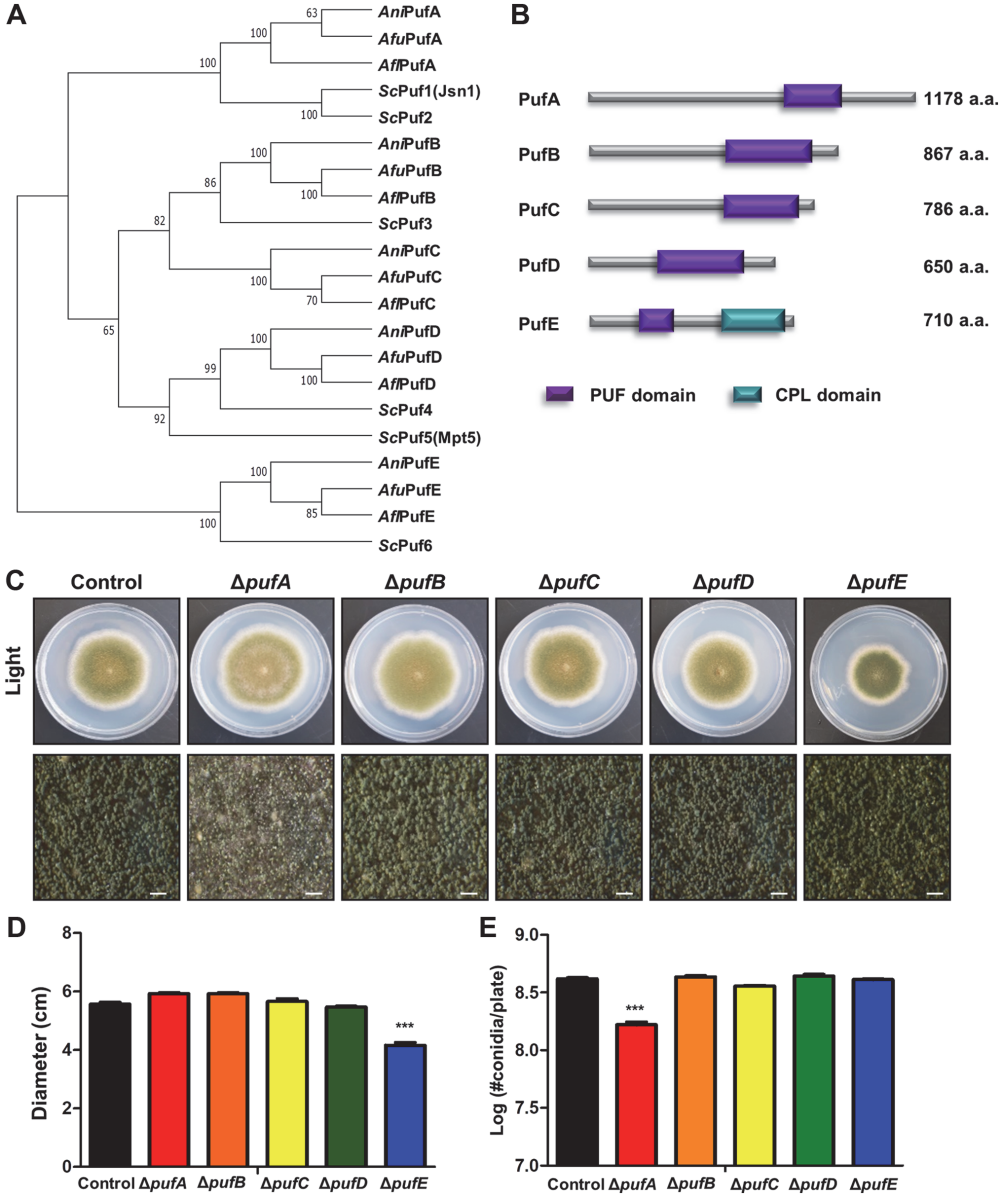
Summary of the *puf* genes in *A. nidulans*. (**A**) Phylogenetic tree of the putative Puf proteins in *A. nidulans* FGSC4, *A. fumigatus* AF293, *A. flavus* NRRL 3357, and *S. cerevisiae* SC288. (**B**) Domain architecture of the putative Puf proteins in *A. nidulans*. (**C**) Control (TNJ36) and *puf* deletion mutant strains grown on MMG agar plates for 5 days at 37°C under light condition. The bottom panel shows close-up views of the center of the colony (bar = 0.25 μm). (**D**) Colony diameter of control (TNJ36) and *puf* deletion mutant strains shown in (A). (**E**) Number of conidia of control (TNJ36) and *puf* deletion mutant strains shown in (A).

Comparative genomic analyses found five genes encoding PUF repeat-containing proteins in three *Aspergillus* species: *A. nidulans*, *A. oryzae*, and *A. fumigatus* [54]. The predicted *Aspergillus* PUF protein sequences were aligned with the *S. cerevisiae* Puf1-Puf6 proteins using Clustal Omega (https://www.ebi.ac.uk/Tools/msa/clustalo/) and MEGA phylogenetic analysis tools. We predicted that AN7474, AN6587, AN4285, AN10071, and AN10173 are the potential homologs of *S. cerevisiae* Puf1 (identity 46.13%), Puf3 (identity 41.49%), Puf3 (identity 26.40%), Puf4 (identity 47.72%), and Puf5 (identity 31.76%), respectively. Based on these results, *A. nidulans* *puf* genes were named *pufA* (AN7474), *pufB* (AN6587), *pufC* (AN4285), *pufD* (AN10071), and *pufE* (AN10173) (Fig. 1A). Domain analysis found that all PUF proteins contain three–eight Pumilio-like repeats (SM00025). PufA, similar to ScPuf1 and ScPuf2, contains six Pumilio-like repeats in a row, and PufB, PufC, and PufD have seven or eight Pumilio-like repeats. Unlike other PUF proteins in *A. nidulans*, PufE contains three Pumilio-like repeats. And PufE has a CPL domain at the C-terminus region, similar to ScPuf6 (Fig. 1B).

To investigate the roles of the five *puf* genes in *A. nidulans*, we generated deletion mutants and examined their colony growth and asexual spore production (Fig. 1C). *pufE* mutants had reduced colony diameter, and *pufA* mutants produced fewer asexual spores than control strain (Figs. 1D and 1E). *pufB*, *pufC*, and *pufD* did not show any growth or sporulation phenotypes. We further analyzed the roles of PufA and PufE proteins in *A. nidulans*.

**Fig. 2:**
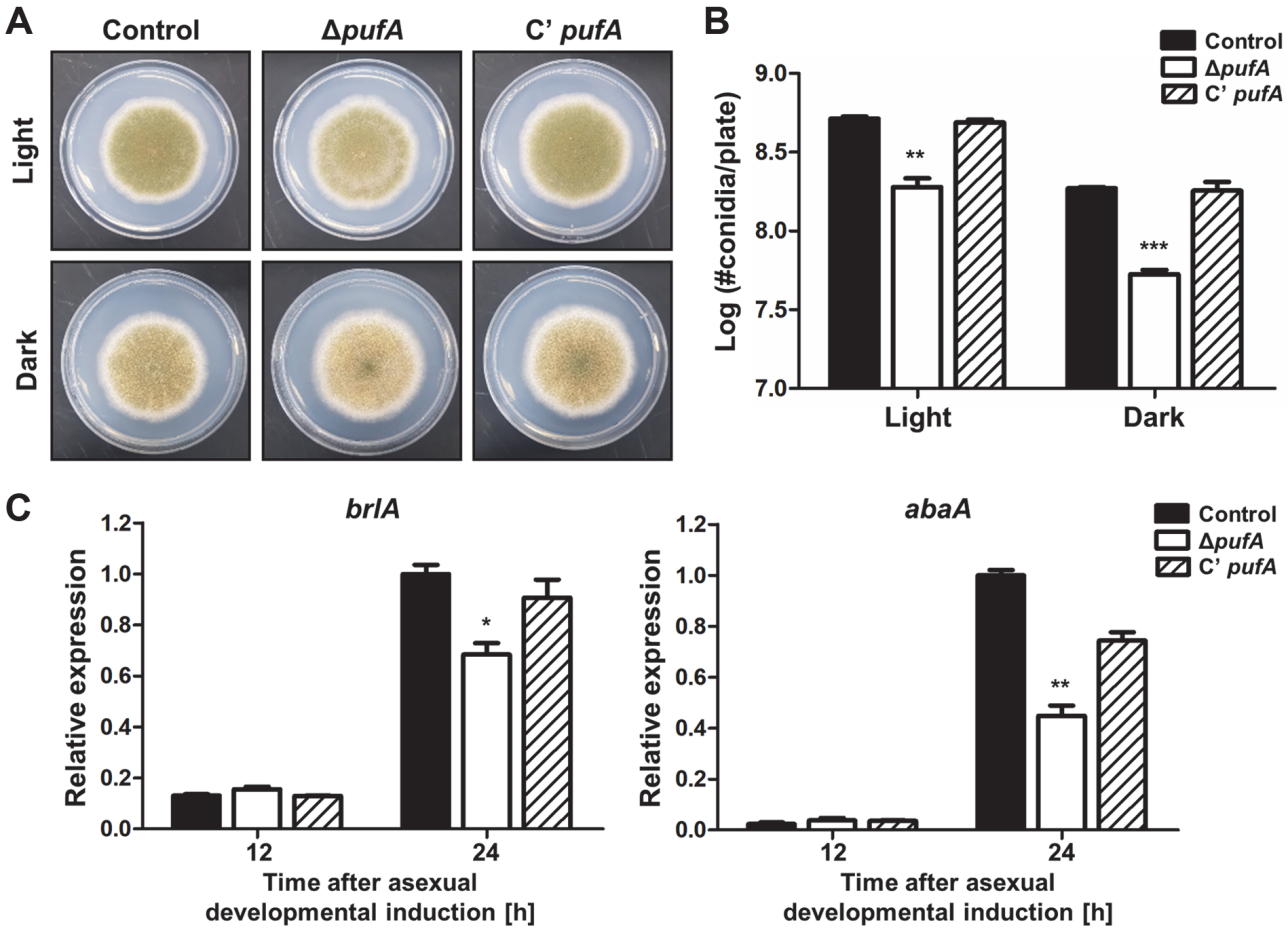
Phenotypic analysis of the Δ*pufA* mutant strains. (**A**) Colony photographs of control (TNJ36), Δ*pufA* (TSH8.1), and complemented *pufA* (C’ *pufA*, TSH17.1) strains point inoculated on solid MMG and grown for 5 days under light or dark conditions. (**B**) Quantitative analysis of asexual spore production of control (TNJ36), Δ*pufA* (TSH8.1), and C’ *pufA* (TSH17.1) strains shown in (A). (**C**) mRNA levels of *brlA* and *abaA* in control (TNJ36), Δ*pufA* (TSH8.1), and C’ *pufA* (TSH17.1) strains shown in (A). Numbers indicate the time (h) after induction of asexual development. Differences between the control and Δ*pufA*, *p < 0.05, **p < 0.01, and ***p < 0.001.

**Deletion of *pufA* Reduces Asexual Spore Formation.** To further examine the role of PufA in asexual development, we generated a *pufA* complemented strain (C’ *pufA*) and compared its phenotype with that of the mutant (Fig. 2A). Similar to what we reported above (Fig. 1), a deletion of *pufA* led to decreased production of asexual spores in both light and dark conditions (Fig. 2B). In addition, deletion of *pufA* affected the mRNA expression of two asexual development regulating genes, *brlA* and *abaA* (Fig. 2C). Transcript levels of *brlA* and *abaA* decreased at 24 h after asexual developmental induction. Asexual spore production and *brlA* and *abaA* mRNA levels in C’ *pufA* were not significantly different from the control. Overall, these results demonstrate that PufA is required for asexual development in *A. nidulans*.

**Fig. 3:**
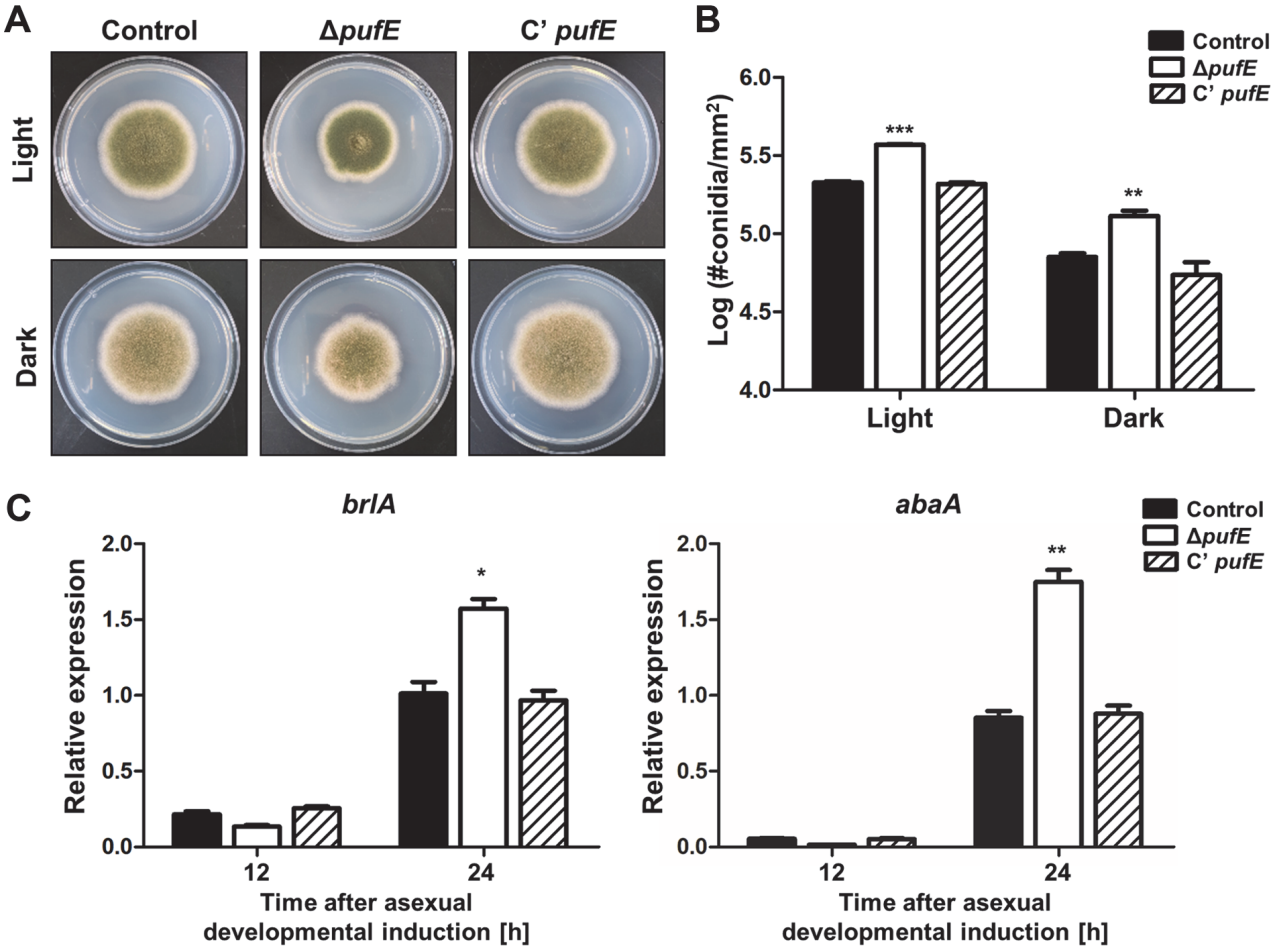
Asexual developmental analysis of the Δ*pufE* mutant strains. (**A**) Colony photographs of control (TNJ36), Δ*pufE* (TSH27.1), and the complemented *pufE* (C’ *pufE*, TSH28.1) strains point inoculated on solid MMG and grown for 5 days under light or dark conditions. (**B**) Quantitative analysis of asexual spore production of control (TNJ36), Δ*pufE* (TSH27.1), and C’ *pufE* (TSH28.1) strains shown in (A). (**C**) mRNA levels of *brlA* and *abaA* in control (TNJ36), Δ*pufE* (TSH27.1), and C’ *pufE* (TSH28.1) strains shown in (A). Numbers indicate the time (h) after induction of asexual development. Differences between the control and Δ*pufE*, *p < 0.05, **p < 0.01, and ***p < 0.001.

**PufE is required for normal asexual and sexual development.** As shown in Fig. 1, the deletion of *pufE* resulted in decreased colony diameter but the number of asexual spores in the entire colony appeared similar to the control. To further understand these phenotypes, we calculated the number of asexual spores per area in mutant and complemented strains. Δ*pufE* had decreased colony diameter but produced more asexual spores than the control and C’ *pufE* strains in both dark and light conditions (Fig. 3). Additionally, the mRNA levels of asexual development-related genes, *brlA* and *abaA*, increased 24 h after asexual developmental induction. These results indicate that PufE is essential for fungal development and asexual development in *A. nidulans*.

**Fig. 4:**
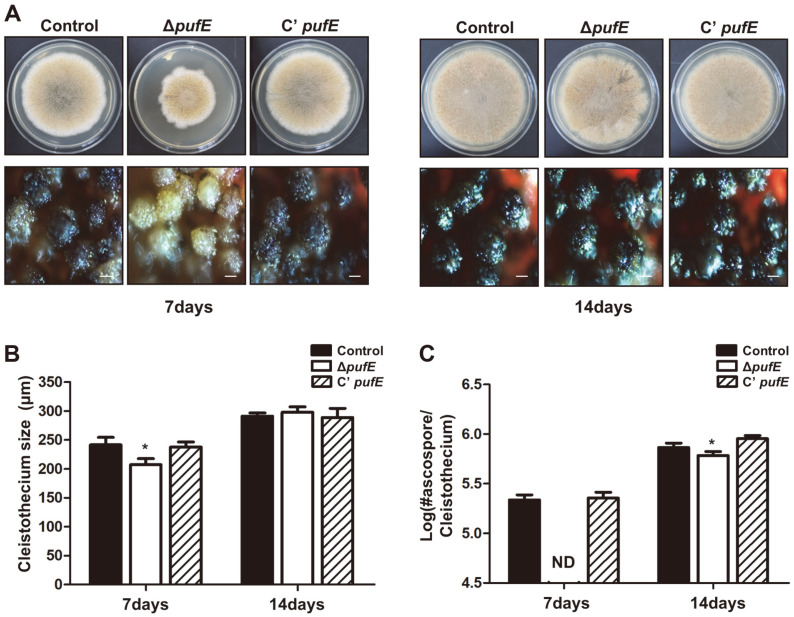
Sexual development of the Δ*pufE* mutant strains. (**A**) Sexual fruiting body formation of control (TNJ36), Δ*pufE* (TSH27.1), and C’ *pufE* (TSH28.1) strains point inoculated on solid SM and grown for 7 or 14 days under dark condition. The bottom panel shows close-up views of the center of the plates (Bar = 200μm). (**B**) Size of cleistothecium of control (TNJ36), Δ*pufE* (TSH27.1), and C’ *pufE* (TSH28.1) strains shown in (A). (**C**) Number of ascospores (log scale) per cleistothecium of control (TNJ36), Δ*pufE* (TSH27.1), and C’ *pufE* (TSH28.1) strains. Differences between the control and Δ*pufE*, *p < 0.05. ND, not detected.

We also examined the role of PufE in sexual development in *A. nidulans* (Fig. 4). Control, Δ*pufE*, and C’ *pufE* strains were point inoculated into SM and incubated under the dark condition. At 7 days after incubation, control and C’ *pufE* strains produced matured sexual fruiting bodies; however, Δ*pufE* sexual fruiting bodies remained immature (Fig. 4A). Δ*pufE* cleistothecia were smaller than in control and C’ *pufE* strains (Fig. 4B). Ascospores were not detected in Δ*pufE* cleistothecium but were present in control and C’ *pufE* strains (Fig. 4C). At 14 days, Δ*pufE* strains produced matured cleistothecia; cleistothecium size was similar in all strains. However, Δ*pufE* cleistothecium contained fewer ascospores than in control and C’ *pufE*. These results indicate that PufE is involved in the formation and maturation of sexual fruiting bodies in *A. nidulans*.

PufE is an ortholog of *S. cerevisiae* Puf6. It contains three PUF repeat motifs and a CPL domain at the C-terminal region. Here, we found that PufE is required for proper fungal development. The Δ*pufE* mutants have increased asexual spore production (Fig. 3) and delayed sexual fruiting body formation (Fig. 4). These results imply that PufE acts as a developmental balancer for asexual and sexual development.

**Fig. 5:**
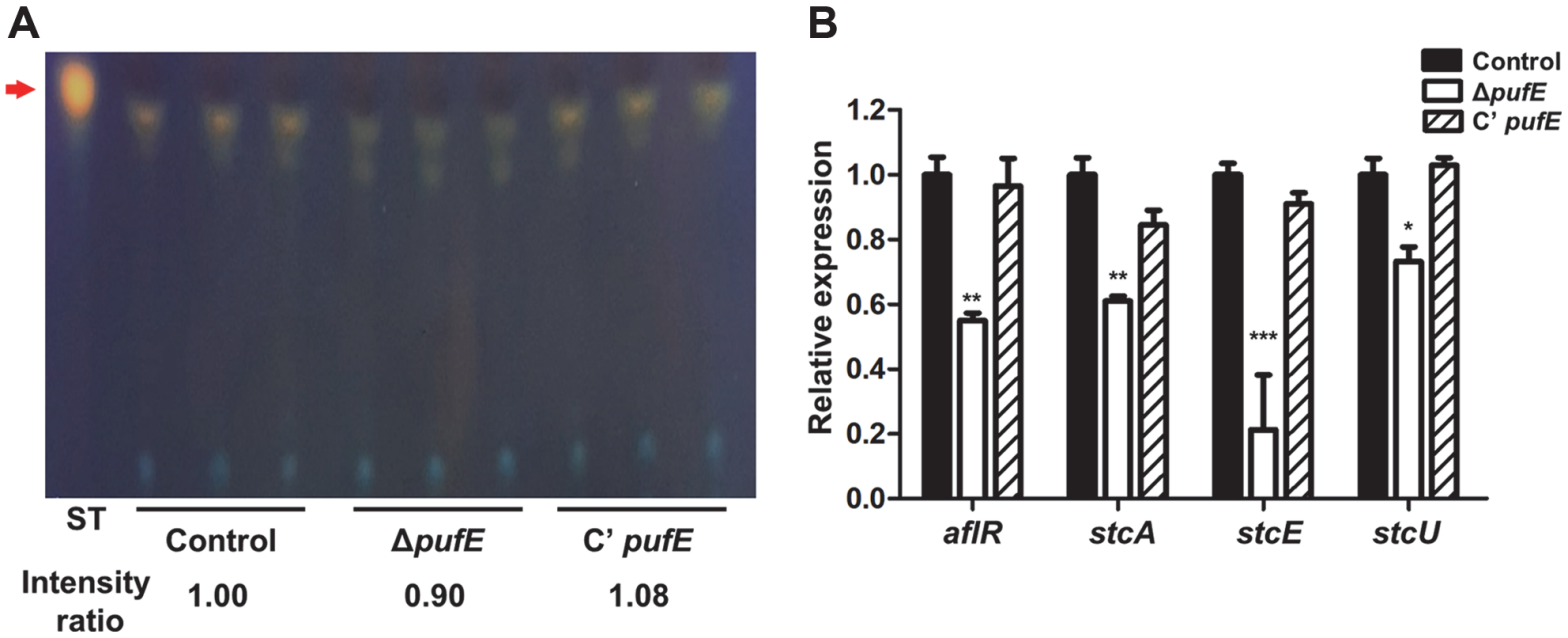
Sterigmatocystin production in the Δ*pufE* mutant strains. (**A**) TLC image of sterigmatocystin from control (TNJ36), Δ*pufE* (TSH27.1), and C’ *pufE* (TSH28.1) strains. These strains were cultured into CM for 7 days under dark condition. Arrow indicates sterigmatocystin for standard. (**B**) mRNA levels of *aflR*, *stcA*, *stcE*, and *stcU* in control (TNJ36), Δ*pufE* (TSH27.1), and C’ *pufE* (TSH28.1). Differences between the control and Δ*pufE*, *p < 0.05, **p < 0.01, and ***p < 0.001.

**Deletion of PufE reduces sterigmatocystin biosynthesis.** We further investigated if PufE also has a role in regulating *A. nidulans* secondary metabolism by analyzing the production of the key secondary metabolite, sterigmatocystin, in Δ*pufE* strains. Sterigmatocystin extracts were separated in a TLC plate. Δ*pufE* mutants produced less sterigmatocystin than control and C’ *pufE* strains (Fig. 5A). To further investigate sterigmatocystin biosynthesis, we checked the mRNA levels of several genes in the sterigmatocystin gene cluster. mRNA levels of *aflR*, *stcA*, *stcE*, and *stcU* were lower in Δ*pufE* than in the control and C’ *pufE* strains (Fig. 5B). These results suggest that PufE is required for normal sterigmatocystin biosynthesis in *A. nidulans*.

### Role of PufE in A nidulans Conidia

**Fig. 6:**
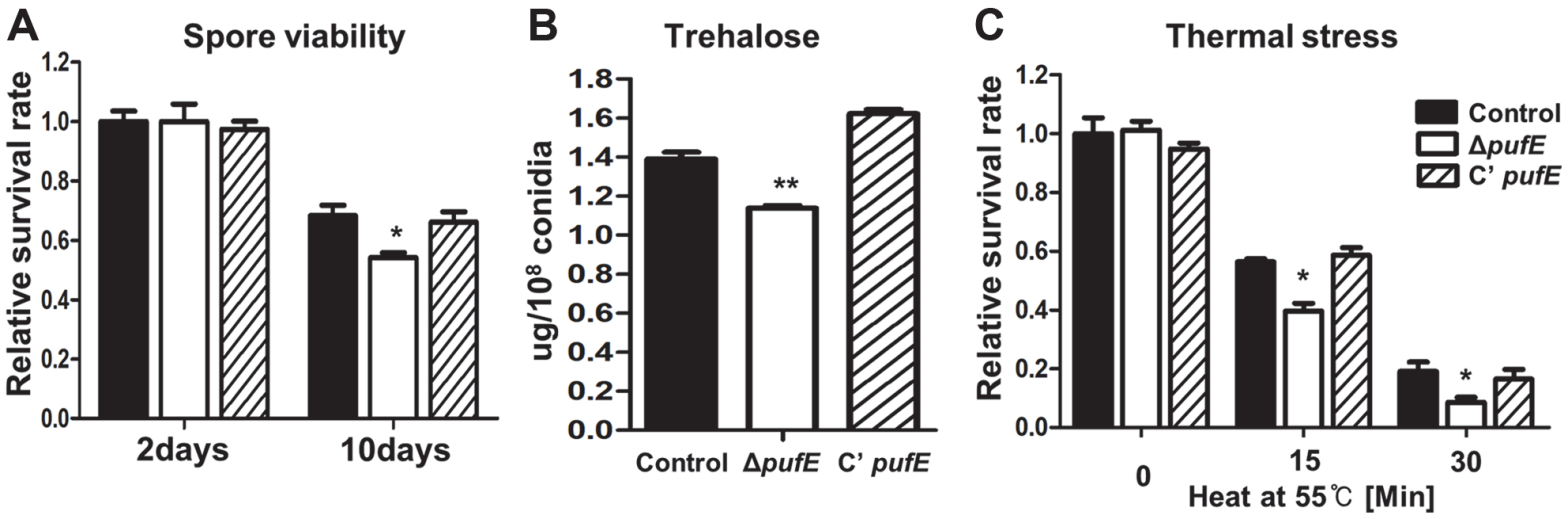
Spore viability and thermal tolerance of the Δ*pufE* mutant conidia. (**A**) Spore viability of control (TNJ36), Δ*pufE* (TSH27.1), and C’ *pufE* (TSH28.1) strains. (**B**) Trehalose contents in the 2-day-old conidia (10^8^) of control (TNJ36), Δ*pufE* (TSH27.1), and C’ *pufE* (TSH28.1) strains (measured in triplicate). (**C**) Heat tolerance of control (TNJ36), Δ*pufE* (TSH27.1), and C’ *pufE* (TSH28.1) conidia (triplicate measurements). Differences between the control and Δ*pufE*, *p < 0.05, **p < 0.01, and ***p < 0.001.

To further understand PufE developmental roles, we examined *pufE* mRNA levels throughout the *A. nidulans* lifecycle (Fig. S1). *pufE* mRNA levels were high in conidia during asexual development, indicating a role in conidia production. To test this hypothesis, we first analyzed the viability of mutant spores. Loss of *pufE* slightly decreased spore viability (Fig. 6A). We then determined conidia trehalose content, a key factor for spore viability and stress tolerance. The *pufE* deletion mutant conidia contained less trehalose than the controls (Fig. 6B). In addition, the *pufE* deletion mutant conidia were more sensitive to heat stress than those from control and C’ *pufE* strains (Fig. 6C). These results suggest that PufE plays an important role in conidial viability and stress tolerance.

### Conclusion

In this study, we investigated the developmental roles of five *A. nidulans* PUF proteins. Two of the tested proteins, PufA and PufE, are necessary for proper asexual and sexual development. In addition, PufE plays a vital role in spore viability, thermal stress response, and sterigmatocystin production. These results indicate that PufA and PufE have important roles in development, similar to other eukaryotic PUF proteins. The function of *A. nidulans* PUF proteins in secondary metabolism and stress responses has been studied, but the *puf* genes do not have a significant effect for stress response and sterigmatocystin production (Figs. S2 and S3). Although the phenotypic analyses were conducted for the *puf* deletion mutants, the RNA-binding motifs and the targets of *A. nidulans* PUF proteins remain largely unknown. Therefore, further studies are needed to elucidate their biological roles and molecular mechanisms in *A. nidulans* development and metabolism.

## Supplemental Materials



Supplementary data for this paper are available on-line only at http://jmb.or.kr.

## Figures and Tables

**Table 1:** *Aspergillus* strains used in this study.

Strain name	Relevant genotype	References
FGSC A4	*A. nidulans* wild type, *veA^+^*	FGSC^a^
RJMP1.59	*pyrG89*; *pyroA4*; *veA*^+^	[55]
TNJ36	*pyrG89;* *AfupyrG* ^+^; *pyroA4*; *veA*^+^	[56]
TSH8.1~3	*pyrG89*; *pyroA4*; *∆pufA*::*AfupyrG^+^*; *veA*^+^	This study
TSH10.1~3	*pyrG89*; *pyroA4*; *∆pufB*::*AfupyrG^+^*; *veA*^+^	This study
TSH11.1~3	*pyrG89*; *pyroA4*; *∆pufC*::*AfupyrG^+^*; *veA*^+^	This study
TSH9.1~3	*pyrG89*; *pyroA4*; *∆pufD*::*AfupyrG^+^*; *veA*^+^	This study
TSH27.1~3	*pyrG89*; *pyroA4*; *∆pufE*::*AfupyrG^+^*; *veA*^+^	This study
TSH17.1~3	*pyrG89*;*pyroA*::*pufA(p)*::*pufA*::FLAG3x::*pyroA^b^*; *∆pufA*::*AfupyrG^+^*; *veA*^+^	This study
TSH28.1~3	*pyrG89*;*pyroA*::*pufE(p)*::*pufE*::FLAG3x::*pyroA^b^*; *∆pufE*::*AfupyrG^+^*; *veA*^+^	This study

^a^Fungal Genetic Stock Center

^b^The 3/4 *pyroA* marker causes targeted integration at the *pyroA* locus.

**Table 2:** Oligonucleotides used in this study.

Name	Sequence (5′ à 3′)^a^	Purpose
OHS0089	GCTGAAGTCATGATACAGGCCAAA	*AfupyrG* Marker_F
OHS0090	ATCGTCGGGAGGTATTGTCGTCAC	*AfupyrG* Marker_R
OHS0842	GGA AGCTGACTACGCGGAA	*pufA*_5' DF
OHS0843	TTCTCCAGCTTTGGCCCT	*pufA*_3' DR
OHS0844	*GGCTTTGGCCTGTATCATGACTTCA* ACGGTCAAAAGCTCACCCC	*pufA*_Rev with *Af-pyrG* tail R
OHS0845	*TTTGGTGACGACAATACCTCCCGAC* GTCGGA ATTCTCCCATCGC	*pufA*_For with *Af-pyrG* tail F
OHS0846	AAACGAAGGACGGACCAACC	*pufA*_nested 5' NF
OHS0847	GTATCCCGACGCTTCGATGA	*pufA*_nested 3' NR
OHS0848	CTGGAGCTATCACACCGTCT	*pufA*_RT_F
OHS0849	CGATTGACAGTCGCATGGTT	*pufA*_RT_R
OHS0834	ATCGCGTCCTCGTCCTATC	*pufB*_5' DF
OHS0835	ATCCTCTAACCGTTCGCGC	*pufB*_3' DR
OHS0836	*GGCTTTGGCCTGTATCATGACTTCA* TTATTGGAGGGACCGACGAC	*pufB*_Rev with *Af-pyrG* tail R
OHS0837	*TTTGGTGACGACAATACCTCCCGAC* GCCTACATCGAGGGTCGTT	*pufB*_For with *Af-pyrG* tail F
OHS0838	ACACAATTCTTGGCTCCCCG	*pufB*_nested 5' NF
OHS0839	CATAGCCTGATGTGCCGCT	*pufB*_nested 3' NR
OHS0840	CTCCTGTCCAGAATCGCTCT	*pufB*_RT_F
OHS0960	ATCCTGATTGTGCCGGGTAA	*pufB*_RT_R
OHS0850	AGCCGCAACCTCCTACAA	*pufC*_5' DF
OHS0851	CCGCTAGATGTTGCGACCT	*pufC*_3' DR
OHS0852	*GGCTTTGGCCTGTATCATGACTTCA* CGATAGGGCACAATGGGCT	*pufC*_Rev with *Af-pyrG* tail R
OHS0853	*TTTGGTGACGACAATACCTCCCGAC* CCGGTA AGACTATTC AGC	*pufC*_For with *Af-pyrG* tail F
OHS0854	GCCTAACGAAGGGTTGGAGT	*pufC*_nested 5' NF
OHS0855	TAAAGACTTGGACCGGGGCT	*pufC*_nested 3' NR
OHS0856	CCAAAGAAGTTGCGCGTTTC	*pufC*_RT_F
OHS0857	CGGGTTTGTGCTAGTTCCTG	*pufC*_RT_R
OHS0826	ATGTTGCGCGGCCTATTG	*pufD*_5' DF
OHS0827	AGCATGCGGTACTTCGAGT	*pufD*_3' DR
OHS0828	*GGCTTTGGCCTGTATCATGACTTCA* CTG ATGAGAAGAGCGGTGACG	*pufD*_Rev with *Af-pyrG* tail R
OHS0829	*TTTGGTGACGACAATACCTCCCGAC* CGAACAACAATCTTGGCGCTC	*pufD*_For with *Af-pyrG* tail F
OHS0830	GCGAAAGCAGGCATATCTGG	*pufD*_nested 5' NF
OHS0831	CACCTCGATCGGCAGCATA	*pufD*_nested 3' NR
OHS0832	ACAACGTCAGCTTGGGTAGA	*pufD*_RT_F
OHS0833	TGCAATGTCGTAGGCTGAGA	*pufD*_RT_R
OHS1330	CAACCCCATTGAGCCTCAG	*pufE*_5' DF
OHS1331	GGGATTAGCACAGCACGTG	*pufE*_3' DR
OHS1332	*GGCTTTGGCCTGTATCATGACTTCA* GTGGCTCCA AGGTCTGTCT	*pufE*_Rev with *Af-pyrG* tail R
OHS1333	*TTTGGTGACGACAATACCTCCCGAC* AGGGCCATCATTTCGCCA	*pufE*_For with *Af-pyrG* tail F
OHS1334	CTCGATTTCCCGTGAGTCTTTCG	*pufE*_nested 5' NF
OHS1335	TCA ATA AGCTCGGACTGCCTG	*pufE*_nested 3' NR
OHS1336	GAACCTGCTTAGGGAGTGGT	*pufE*_RT_F
OHS1337	TTCGAGGAGGAAGTGCATGA	*pufE*_RT_R
OHS1136	AATT**GCGGCCGC**ACGGTACACACTTTACCCGG	C’ *pufA*_Not1F
OHS1137	AATT**GCGGCCGC**GAAGGTATAGGCAAAGTTCAGCGC	C’ *pufA*_Not1R
OHS1380	AATT**GCGGCCGC**GAAATCGCCATATGCGCC	C’ *pufE*_Not1F
OHS1381	AATT**GCGGCCGC**CGCAATCTTCTCCAACAATAACTT	C’ *pufE*_Not1R
OHS0580	CAAGGCATGCATCAGTACCC	*brlA*_RT_F
OHS0581	AGACATCGAACTCGGGACTC	*brlA*_RT_R
OHS0779	ATTGACTGGGAAGCGAAGGA	*abaA*_RT_F
OHS0780	CTGGGCAGTTGAACGATCTG	*abaA*_RT_R
OHS0599	GCGCGAAGAAGACTTCAAC	*aflR*_RT_F
OHS0600	TGCAATAACTGCCGACGAC	*aflR*_RT_R
OHS0946	GGATCTGCCAAAGCGAACAT	*stcA*_RT_F
OHS0947	CCACAGTGAGGAGGAATGGT	*stcA*_RT_R
OHS0604	GCTACTGTTCCAGGCGACTA	*stcE*_RT_F
OHS0605	CACAGCTCTCCATCTCGGTA	*stcE*_RT_R
OHS0602	CGCATCATCCTCACAAGTTC	*stcU*_RT_F
OHS0603	TGACCGTGATCTTCTTGTCG	*stcU*_RT_R

^a^Tail sequences are shown in italics. Restriction enzyme sites are in bold.
