# Supplementary material for: Functions of PUF Family RNA-Binding Proteins in Aspergillus nidulans
Source: J Microbiol Biotechnol. 2021 Mar 12;31(5):676–85. doi: 10.4014/jmb.2101.01011 (PMC9706018; doi:10.4014/jmb.2101.01011)
Supplement: Supplementary file 1 [file jmb-31-5-676-supple.pdf]

J. Microbiol. Biotechnol.
https://doi.org/10.4014/jmb.2101.01011

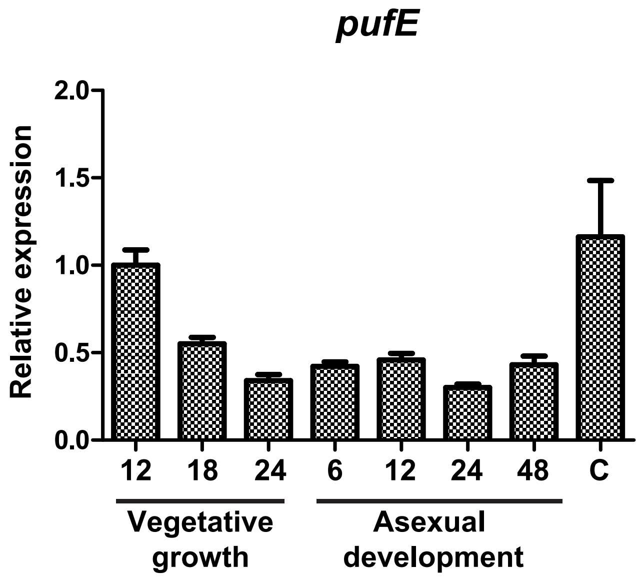

Figure S1. mRNA levels of *pufE* during the lifecycle of *A. nidulans.* Conidia (asexual spores) are indicated as C. The time (hours) of incubation in vegetative growth and post asexual developmental induction is shown.

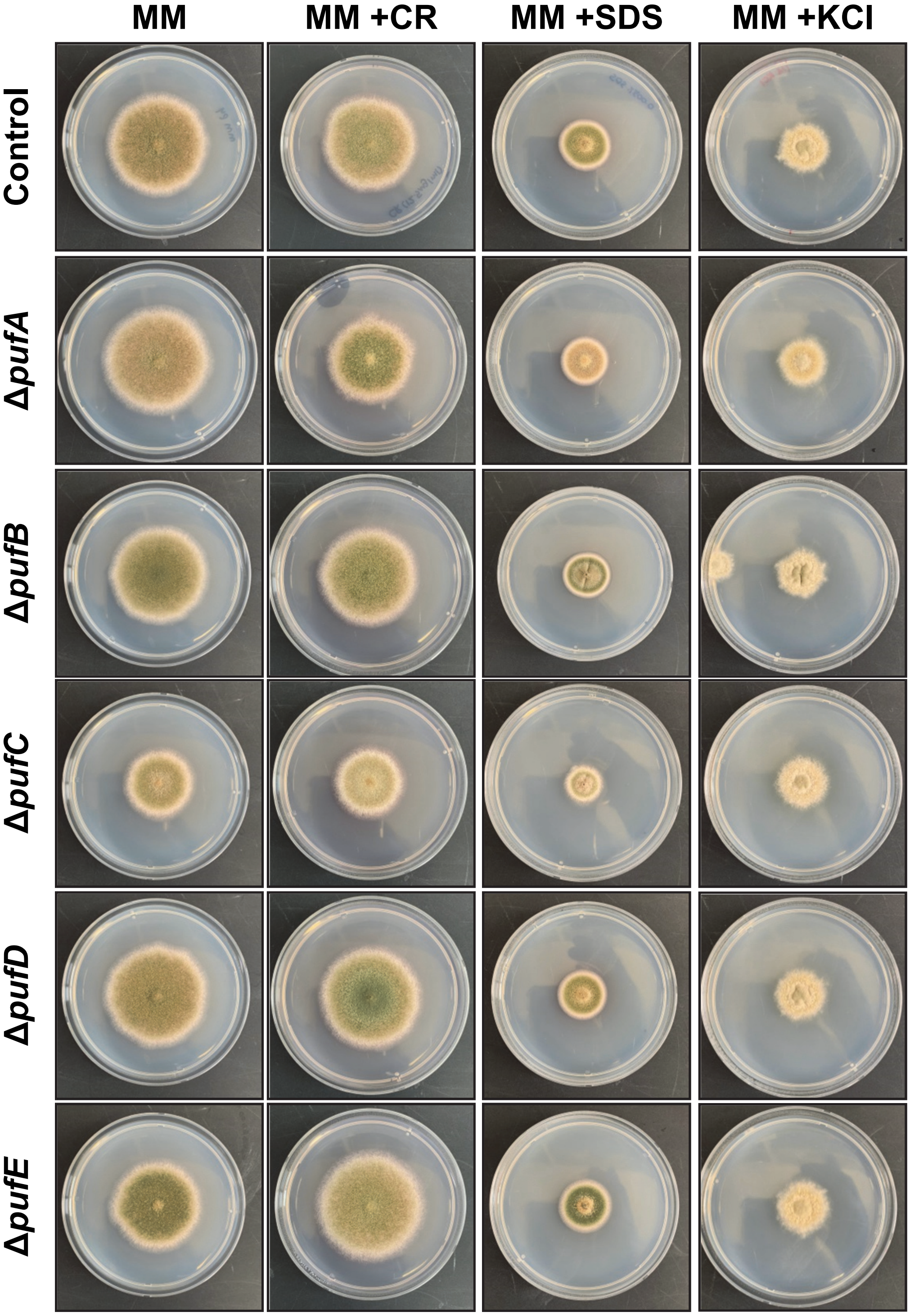

Figure S2. Sensitivity of *puf* mutant strains to various stress conditions. Control and *puf* mutant strains were inoculated on solid MM medium containing various compounds including 12.5㎕/mL Congo Red (CR), 0.005% SDS, and 1M KCl.

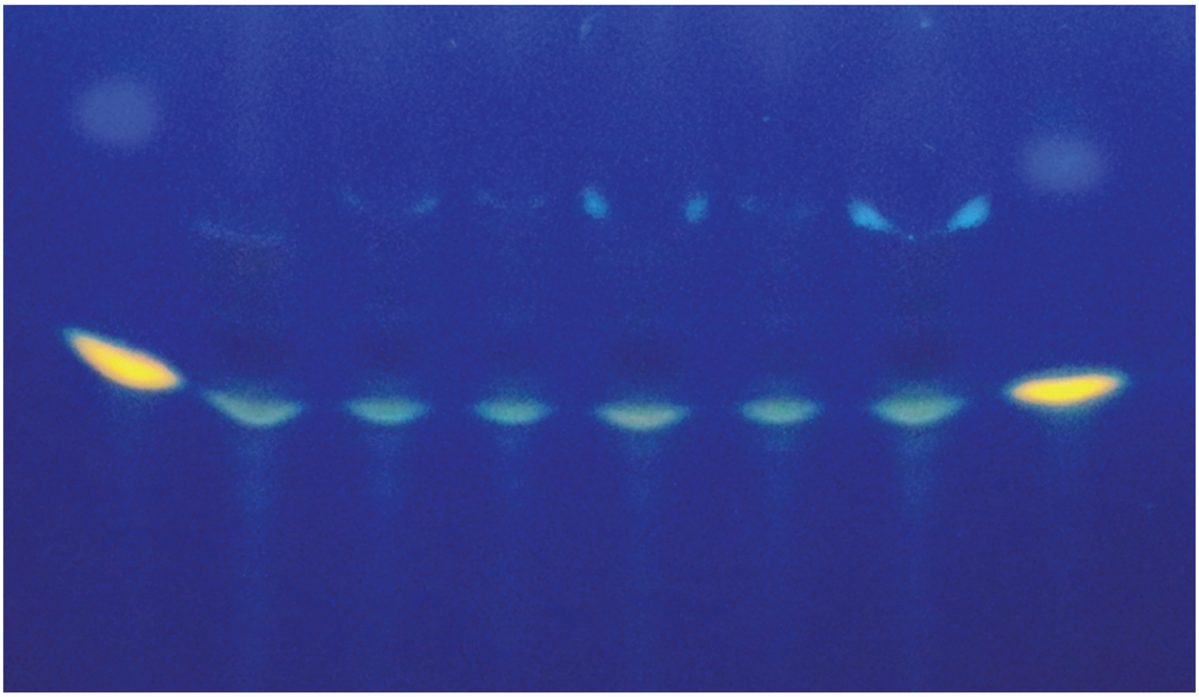

Figure S3. Sterigmatocystin production in the *puf* mutant strains. Thin-layer chromatography (TLC) analysis of sterigmatocystin (ST) produced by control and the *puf* mutant strains.
